# Prognostic value of endothelial biomarkers in refractory cardiogenic shock with ECLS: a prospective monocentric study

**DOI:** 10.1186/s12871-019-0747-1

**Published:** 2019-05-15

**Authors:** Tsung-Yu Tsai, Kun-Hua Tu, Feng-Chun Tsai, Yu-Yun Nan, Pei-Chun Fan, Chih-Hsiang Chang, Ya-Chung Tian, Ji-Tseng Fang, Chih-Wei Yang, Yung-Chang Chen

**Affiliations:** 1 College of Medicine, Chang Gung University, Taoyuan, Taiwan; 2 Department of Nephrology, Kidney Research Center, Chang Gung Memorial Hospital, Taoyuan, Taiwan; 3 Division of Cardiovascular Surgery, Chang Gung Memorial Hospital, Taoyuan, Taiwan; 4 Department of Nephrology, Chang Gung Memorial Hospital, Chang Gung University College of Medicine, No.222, Maijin Road, Anle District, Keelung, Taiwan

**Keywords:** Extracorporeal membrane oxygenation, Angiopoietin, Vascular endothelial growth factor, Endothelial biomarker

## Abstract

**Background:**

Extracorporeal membrane oxygenation (ECMO) is often used in critical patients with severe myocardial failure. However, the mortality rate of patients on ECMO is often high. Recent studies have suggested that endothelial activation with subsequent vascular barrier breakdown is a critical pathogenic mechanism of organ damage and is related to the outcome of critical illness. This study aimed to determine whether endothelial biomarkers can be served as prognostic factors for the outcome of patients on ECMO.

**Methods:**

This prospective study enrolled 23 critically ill patients on veno-arterial ECMO in the intensive care units of a tertiary care hospital between March 2014 and February 2015. Serum samples were tested for thrombomodulin, angiopoietin (Ang)-1, Ang-2, and vascular endothelial growth factor (VEGF). Demographic, clinical, and laboratory data were also collected.

**Results:**

The overall mortality rate was 56.5%. The combination of Ang-2 at the time of ECMO support (day 0) and VEGF at day 2 had the ability to discriminate mortality (area under receiver operating characteristic curve [AUROC], 0.854; 95% confidence interval: 0.645–0.965).

**Conclusions:**

In this study, we found that the combination of Ang-2 at day 0 and VEGF at day 2 was a modest model for mortality discrimination in this group of patients.

## Introduction

Extracorporeal membrane oxygenation (ECMO) is often used in critical patients with severe myocardial failure (e.g., cardiogenic shock or myocarditis). It provides these patients with temporary circulatory support and has been utilized as a bridging therapy for further treatment. However, despite the rapid advances in the ECMO technique and post-operative care in recent decades, the mortality rate of patients on ECMO remains high [1–4].

Previous studies have shown that several intensive care unit (ICU) scoring systems have good ability in outcome prediction for patients on ECMO [1, 5, 6]. However, these scoring systems usually consist of many laboratory data and physiological measurements, and sometimes need complex calculation. Recently, several biomarkers have been applied to predict renal and neurologic outcomes in patients on ECMO [7, 8], but no particular biomarker is associated with mortality in this patient group.

Recent studies have shown that endothelial activation with subsequent vascular barrier breakdown is a critical pathogenic mechanism of organ damage and is related to the outcome of critical illness [9–12]. Thrombomodulin (TM) is a transmembranous glycoprotein found on the vascular endothelium [13]. It enhances thrombin-induced protein C activation and has roles in inflammation, coagulation, and fibrinolysis [14]. Soluble thrombomodulin levels are associated with mortality in patients with disseminated intravascular coagulation, sepsis, or acute respiratory distress syndrome [12, 15, 16]. Angiopoietin (Ang)-1, Ang-2, and vascular endothelial growth factor (VEGF) are proteins associated with angiogenesis. Ang-1 has an anti-inflammatory effect by limiting endothelium activation, while Ang-2 triggers an inflammatory response by activating the endothelium. Besides, Ang-1 downregulates VEGF expression and reduces thrombin-induced permeability [17–19]. In recent studies, low Ang-1 concentration and high Ang-2 concentration are associated with increased mortality in patients with sepsis [10, 20–22]. However, the relationship between VEGF level and mortality is discordant in different studies [12, 23, 24].

Although endothelial activation and injury are involved in organ damage and associated with the prognosis of critical illness, there has been no associated study on patients on ECMO. Therefore, this study aimed to determine whether the serum biomarkers of endothelial injury and activation could serve as prognostic factors for the outcome of patients on ECMO.

## Materials and methods

### Study population and data collection

The local Institutional Review Board (IRB) of Chang Gung Memorial Hospital approved the study protocol (IRB No. 103-1569C). The study was performed in the ICUs of a tertiary care hospital in Taiwan between March 2014 and February 2015. Patients who met the inclusion criteria were invited to participate in the study on the first day of ECMO support. Written informed consent was obtained from the next-of-kin of the patients before their participation. The following patients were excluded: pediatric patients younger than 18 years old, those with end stage renal disease undergoing regular renal replacement therapy, and those whose next-of-kin declined study enrollment. Besides, patients with veno-venous (V-V) ECMO support were also excluded due to different pathophysiologic changes between veno-arterial (V-A) and V-V ECMO. For patients with repeated ECMO support during hospitalization, we only collected the data on the first ECMO support. A total of 66 patients were screened during the study period, but the next-of-kin of 43 patients refused consent due to the critical condition of the patients. In total, 23 patients were enrolled.

The following data were prospectively collected: demographic data, indications for ECMO support, and outcomes. We utilized the worst physiological values on the day of ECMO support for physiological calculations. The primary study outcome was in-hospital mortality. Follow-up was performed at 6 months after hospital discharge via chart records or telephone interviews if necessary.

### Sampling and quantifying serum biomarkers

Ten milliliters of blood were collected from each patient with routine blood tests performed at the time of ECMO support (day 0), the morning of the first post-ECMO day (day 1), and the morning of the second post-ECMO day (day 2). The blood samples were centrifuged at 1000 g for 5 min, and the supernatants were stored at − 80 °C. Serum biomarkers (Ang-1, Ang-2, VEGF, and TM) were quantified by an enzyme-linked immunosorbent assay (R&D system, Minneapolis, MN, USA) according to manufacturer instructions.

### Clinical management

The ECMO device (Medtronic, Inc., Anaheim, CA) consisted of a centrifugal pump and a hollow-fiber microporous membrane oxygenator with an integrated heater. All ECMO circuits had a heparin-bound Carmeda bioactive surface. A silicone oxygenator (Medtronics, Minneapolis, MN, USA) was incorporated into the ECMO circuit. A 17–19 Fr percutaneous arterial (outflow) cannula and a 19–21 Fr percutaneous venous (inflow) cannula (DLP; Medtronic Inc., Minneapolis, MN) were chosen according to patients’ body size. Percutaneous access through the common femoral vein (inflow) and the common femoral artery (outflow) was preferred for V-A ECMO. If cyanosis was noted on the cannulated limb, an 8 Fr distal perfusion catheter would be implanted into the ipsilateral superficial femoral artery.

### Statistical analysis

There was no sufficient power to test normality of continuous variables due to the small sample size of this study. Therefore, all statistical tests were done using nonparametric statistics. Descriptive statistics for continuous variables were expressed as median with interquartile range. Data between the survivors and non-survivors were compared using Mann-Whitney U test for continuous variables or Fisher’s exact test for categorical variables. The performance of discriminating mortality by those biomarkers at day 0, day 1, and day 2 of ECMO support was assessed using receiver operating characteristic (ROC) curve analysis. All statistical tests were two-tailed, and a value of *P* < 0.05 was considered statistically significant. No adjustment for multiple testing (multiplicity) was made in this study. Statistical analysis was conducted using SPSS 22 software (IBM SPSS, Armonk, NY: IBM Corp).

## Results

**Fig. 1:**
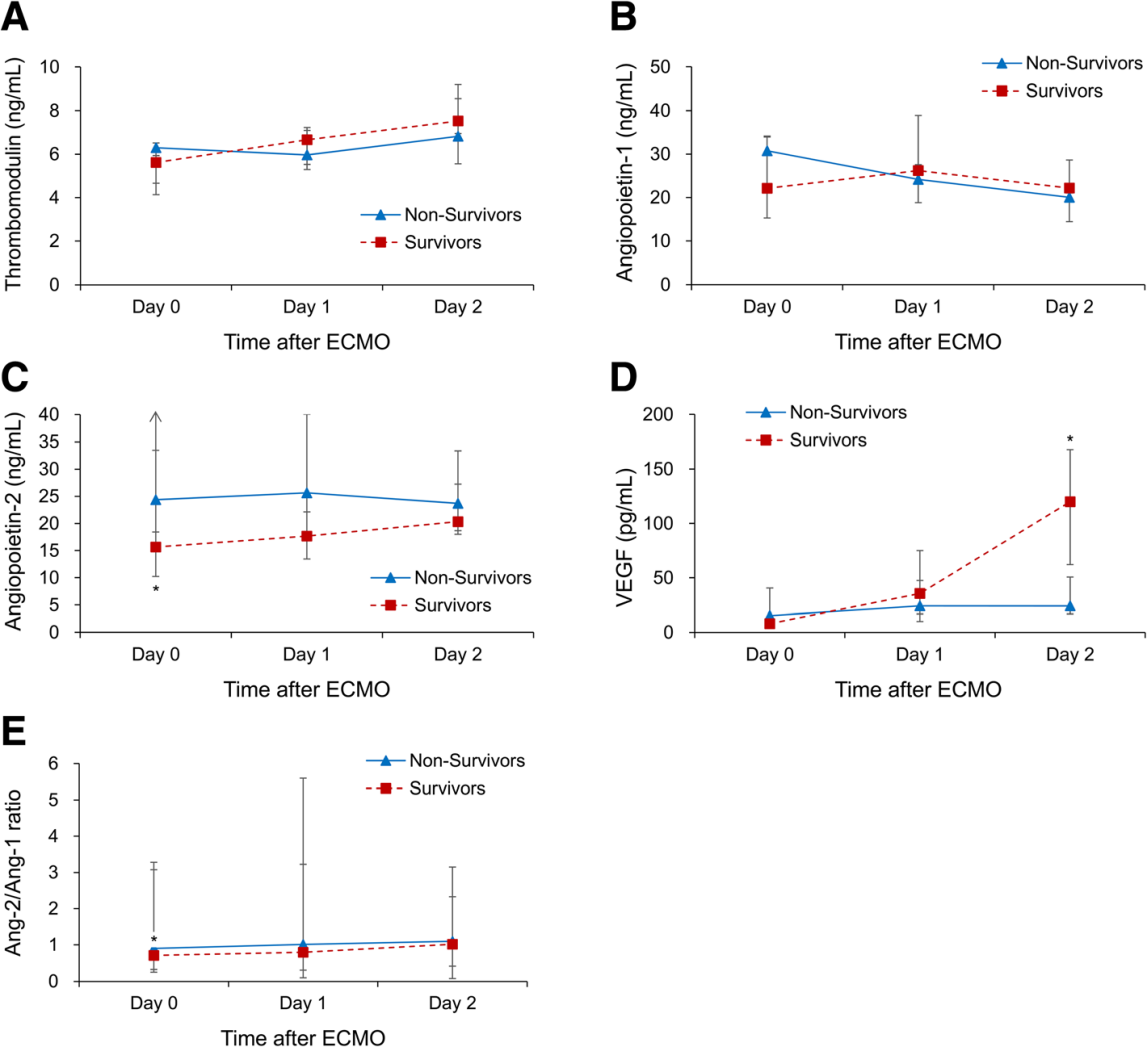
Median values (lower limit of bar represents 25th percentile and upper limit of bar represents 75th percentile) of endothelial biomarkers in the non-survivors and survivors. * indicates *P* < 0.05 between non-survivors and survivors. ECMO, extracorporeal membrane oxygenation; VEGF, vascular endothelial growth factor; Ang, angiopoietin

**Fig. 2:**
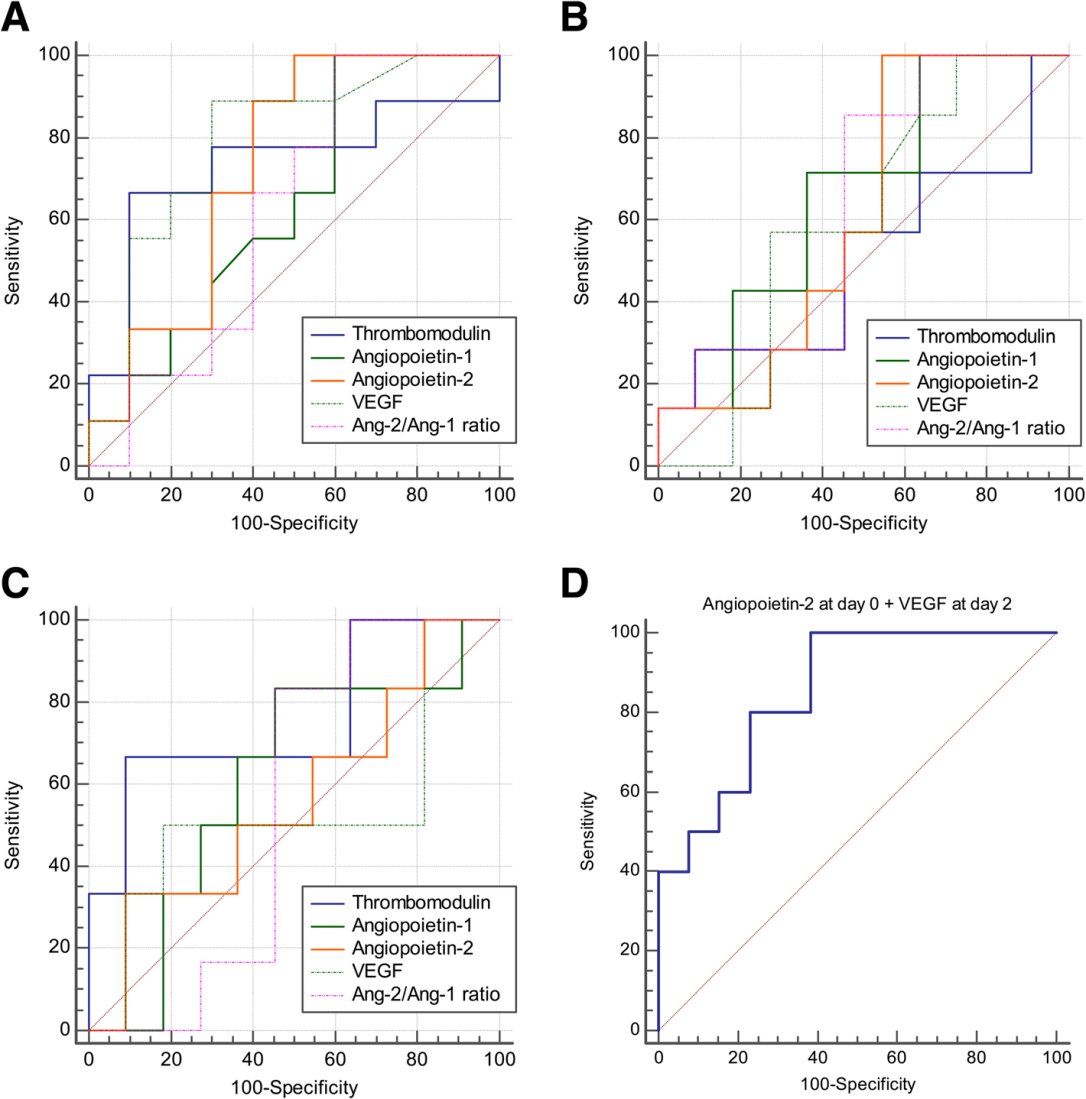
Receiver operating characteristic curves (ROC) of discriminating mortality for (**a**) at day 0, (**b**) at day 1, (**c**) at day 2, and (**d**) combination of angiopoietin-2 at day 0 and VEGF at day 2. The area under ROC of angiopoietin-2 at day 0 + VEGF at day 2 was 0.854 (95% confidence interval, 0.645 to 0.965). VEGF, vascular endothelial growth factor; Ang, angiopoietin

Between March 2014 and February 2015, 23 patients on ECMO support at the ICU were enrolled. The average age was 57 years and 19 (82.6%) were male. The in-hospital mortality rate was 56.5% (13/23). Table 1 presents the demographic data and clinical characteristics of the patients. Non-survivors had higher vasopressor/inotrope dose and higher Sequential Organ Failure Assessment (SOFA) score than survivors at the day of ECMO supplement. Table 2 shows the concentration changes of biomarkers at day 0, day 1, and day 2 of ECMO support. TM and Ang-1 concentrations showed no significant difference between survivors and non-survivors during the first 2 days. The Ang-2/Ang-1 ratio increased gradually in both groups and was higher in non-survivors. Notably, Ang-2 level decreased at day 0 (median: 15.7 vs. 24.4 ng/mL, *P* = 0.035) and VEGF level tremendously increased at day 2 (median: 119.9 vs. 24.2 pg/mL, *P* = 0.005) in the survivors as compared to non-survivors (Fig. 1). Figure 2 depicts the ROC curves of the four biomarkers in discriminating mortality at day 0, day 1, and day 2 of ECMO support. We found that the combined predictive probability of Ang-2 at day 0 and VEGF at day 2 had the ability of discriminating mortality (area under the ROC curve, 0.854; 95% confidence interval [CI], 0.645–0.965; as shown in Fig. 2d).

**Table 1:** Patients’ demographic data and clinical characteristics

Variable	All Patients (*n* = 23)	Non-Survivors (*n* = 10)	Survivors (*n* = 13)	*P* value
Age (years)	57 (19)	55 (7)	58 (20)	0.250
Male sex, n (%)	19 (82.6)	7 (70)	12 (92.3)	0.281
Diabetes mellitus, n (%)	4 (17.4)	1 (10)	3 (23.1)	0.604
Coronary artery disease, n (%)	15 (65.2)	5 (50)	10 (76.9)	0.221
Duration of ECMO support (days)	5 (5)	7 (11)	4 (1)	0.483
Duration of ICU stay (days)	11 (10)	8 (11)	17 (38)	0.020
Mechanical ventilation (days)	8 (8)	8 (11)	8 (8)	0.454
IABP, n (%)	18 (78.3)	8 (80)	10 (76.9)	1.000
Myocardial failure during operation	10 (55.6)	6 (75)	4 (40)	0.188
Cardiogenic shock	8 (44.4)	2 (25)	6 (60)	0.188
Indication for ECMO, n (%)				0.119
Postcardiotomy	12 (52.2)	5 (50)	7 (53.8)	
Myocarditis	1 (4.3)	1 (10)	0 (0)	
Acute myocardial infarction	6 (26.1)	1 (10)	5 (38.5)	
Heart transplantation	1 (4.3)	1 (10)	0 (0)	
Profound shock with desaturation	2 (8.7)	2 (20)	0 (0)	
VT with cardiogenic shock	1 (4.3)	0 (0)	1 (7.7)	
Complication of ECMO, n (%)				
Lower extremity ischemia	2 (8.7)	1 (10)	1 (7.7)	1.000
Stroke	1 (4.3)	1 (10)	0 (0)	0.435
Coma or brain hypoxia	4 (17.4)	4 (40)	0 (0)	0.024
Significant bleeding	8 (34.8)	4 (40)	4 (30.8)	0.685
Rethoractomy for bleeding	5 (21.7)	2 (20)	3 (23.1)	1.000
Vasopressor/inotrope on ECMO 1st day				
Dopamine (μg/kg/min)	0.0 (9.5)	0.0 (4.7)	0.0 (10.7)	0.538
Norepinephrine (μg/kg/min)	0.1 (0.2)	0.1 (0.3)	0.0 (0.2)	0.324
Dobutamine (μg/kg/min)	0.0 (6.3)	5.0 (5.0)	0.0 (0.0)	0.032
Epinephrine (μg/kg/min)	0.1 (0.4)	0.4 (0.4)	0.0 (0.2)	0.027
Biochemistry data on ECMO 1st day				
MAP (mmHg)	58 (19)	55 (21)	59 (14)	0.306
Diuresis (ml/kg/hr)	0.9 (1.1)	1.2 (1.0)	0.9 (1.0)	0.495
SCr (mg/dL)	1.4 (0.8)	1.3 (0.5)	1.5 (0.9)	0.321
WBC count (cu/mm) × 1000	16.0 (17.8)	16.9 (12.3)	15.7 (17.8)	0.756
Hemoglobin (g/dL)	9.2 (1.5)	9.1 (1.1)	9.4 (2.0)	0.710
Platelets (× 10^9^/L)	9.7 (8.6)	9.0 (7.9)	10.2 (10.9)	0.535
Sodium (mEq/L)	143 (18)	147 (20)	143 (11)	0.456
Potassium (mEq/L)	3.2 (1.8)	3.2 (2.0)	3.6 (1.5)	0.926
Albumin (g/L)	2.7 (0.7)	2.8 (0.2)	2.7 (1.1)	1.000
Lactate (mmol/L)	79.4 (48.9)	83.2 (75.2)	75.3 (15.2)	0.710
PaO2/FiO2	384 (235)	187 (411)	395 (99)	0.193
AaDO2	237 (163)	388 (382)	235 (87)	0.172
APACHE II score	23 (10)	26 (10)	23 (8)	0.153
SOFA score	10 (5)	11 (5)	9 (2)	0.026
Acute kidney injury, n (%)	18 (78.3)	8 (80)	10 (76.9)	1.000
KDIGO criteria (Stage 0/1/2/3)	5/10/4/4	2/4/3/1	3/6/1/3	0.572
Renal replacement therapy, n (%)	10 (43.5)	4 (40)	6 (46.2)	1.000

Continuous data were presented median (interquartile); *ECMO* extracorporeal membrane oxygenation, *ICU* intensive care unit, *IABP* intraaortic balloon pumping, *VT* ventricular tachycardia, *MAP* mean arterial pressure, *SCr* serum creatinine, *WBC* white blood cell, *PaO2* partial pressure of oxygen, *FiO2* fraction of inspired oxygen, *AaDO2* alveolar-arterial oxygen tension difference, *APACHE II* acute physiology and chronic health evaluation II, *SOFA* sequential organ failure assessment, *KDIGO* kidney disease improving global outcomes

**Table 2:** Patients’ endothelial biomarkers in the first 3 days

Biomarker	All Patients (*n* = 23)	Non-Survivors (*n* = 10)	Survivors (*n* = 13)	*P* value
Thrombomodulin (ng/mL)				
Day 0	5.9 (2.4)	6.3 (1.9)	5.6 (1.8)	0.420
Day 1	6.3 (1.9)	6.0 (1.9)	6.7 (1.6)	0.535
Day 2	7.5 (2.7)	6.8 (3.0)	7.5 (2.2)	0.215
Angiopoietin-1 (ng/mL)				
Day 0	29.0 (16.1)	30.8 (13.0)	22.1 (18.7)	0.203
Day 1	24.9 (17.1)	24.2 (8.8)	26.2 (15.8)	0.107
Day 2	20.7 (13.8)	20.1 (6.6)	22.2 (9.5)	0.172
Angiopoietin-2 (ng/mL)				
Day 0	19.2 (24.8)	24.4 (58.2)	15.7 (23.2)	0.035
Day 1	24.7 (35.3)	25.6 (29.0)	17.7 (26.6)	0.137
Day 2	22.7 (15.4)	23.7 (14.7)	20.3 (9.2)	0.577
VEGF (pg/mL)				
Day 0	8.5 (13.7)	15.3 (32.4)	7.9 (2.1)	0.071
Day 1	33.0 (65.0)	24.2 (37.6)	35.6 (58.1)	0.438
Day 2	62.1 (119.2)	24.2 (33.9)	119.9 (105.8)	0.005
Ang-2/Ang-1 ratio				
Day 0	0.82 (1.82)	0.90 (1.53)	0.71 (2.20)	0.470
Day 1	1.01 (1.74)	1.01 (3.67)	0.79 (1.95)	0.342
Day 2	1.09 (0.64)	1.10 (0.21)	1.02 (1.54)	0.763

Data were presented median (interquartile); *VEGF* vascular endothelial growth factor, *Ang* angiopoietin

## Discussion

To our knowledge, this study is the first to investigate the relationship between endothelial biomarkers and mortality in patients on ECMO. In this study, we noticed a higher level of Ang-2 in non-survivors compared to that in survivors. Besides, we also observed that the combination of Ang-2 at day 0 and VEGF at day 2 showed a modest performance on mortality discrimination in patients on ECMO.

The initiation of ECMO brings an immediate and complex inflammatory reaction in patients, as seen in systemic inflammatory response syndrome. The inflammatory reaction then results in the widespread activation of the endothelium and induces pro-inflammatory cytokines secretion [25]. Moreover, active diseases that require ECMO support may be associated with endothelial inflammation, such as cardiotomy surgery and acute myocardial infarction. Non-pulsatile flow during aortic cross-cramping during cardiotomy is associated with diminished endothelial shear stress and reduced endothelial nitrogen oxide production, while intra-aortic balloon pump support provides steady pulsatile flow that induces a steady shear stress on the endothelial cells, thereby reducing endothelial activation and inflammatory response [26, 27]. Acute kidney injury following ECMO support is also related to endothelial injury [28]. Therefore, endothelial injury is an important issue in patients on ECMO.

Previous studies have shown that Ang-2 levels are associated with mortality in critically-ill patients [29–32]. Ang-2, a competitive antagonist of Ang-1, reacts with Tie2 receptor to maintain vascular stability. Upon inflammatory stimuli, Ang-2 is released from the Weibel-Palade bodies, causing capillary leakage and facilitating leukocyte migration [33]. In patients on ECMO, Ang-2 increases in response to early endothelial activation. Although it didn’t reveal a close relationship with acute kidney injury in our study, it still provided a potential marker for mortality prediction in patients on ECMO. Besides, Ang-2/Ang-1 ratio increases during capillary endothelial damage, and high Ang-2/Ang-1 ratio is related to poor outcome in patients with sepsis [20, 21]. In our study, the Ang-2/Ang-1 ratio increased gradually in both groups and was higher in non-survivors, which may implicate more severe endothelial damage in the non-survivor group.

VEGF is considered as an endothelial survival factor that prevents microvascular apoptotic cell loss in vitro [34]. Both low and high VEGF concentrations have been reported in critically-ill patients [24, 30, 35], and the significance of which is not fully understood. In our study, the VEGF concentration in the survivor group continued to increase over the first 72 h and was higher than the non-survivor group, which was similar to previous studies [24]. VEGF modulates the effect of Ang-2 in a context-dependent fashion: Ang-2 promotes basal lamina remodeling and endothelial cell proliferation at high VEGF concentration, but causes endothelial cell death and vessel regression if VEGF is inhibited [36]. In our study, we observed that survivors had significantly higher 72-h VEGF concentration compared to non-survivors. Higher VEGF concentration may modulate the Ang-2 effect and help endothelial cell proliferation and neovascularization, but the detailed relationship with mortality needs further studies to evaluate and confirm.

There are some limitations in our study. First, our study was performed at a tertiary care center with a small sample size. Although it was a prospective study, many next-of-kin of the patients declined to join the study at the time of ECMO support due to the critical condition of the patients. Large-scale studies at multiple centers should be performed to confirm these findings. Second, although we excluded patients on V-V ECMO support and only collected patients on V-A ECMO support, the diversity of the diseases indicated for ECMO support may still affect the results, and further subgroup investigations are needed to explore the relationship between specific diseases and endothelial biomarkers. Third, we did not compare the differences in endothelial biomarker levels with a control group because we could not find a group of patients with the same disease severity but without ECMO support.

In summary, we presented a relationship between endothelial biomarker changes and mortality in patients on V-A ECMO. The combination of Ang-2 at day 0 and VEGF at day 2 was a modest model for mortality discrimination in this group of patients. However, further larger studies are warranted due to the small sample size at a single tertiary-care medical center in this study.

## Abbreviations

Ang: Angiopoietin
AUROC: Area under receiver operating characteristic curve
CI: Confidence interval
ECMO: Extracorporeal membrane oxygenation
ICU: Intensive care unit
IRB: Institutional review board
ROC: Receiver operating characteristic
SOFA: Sequential organ failure assessment
TM: Thrombomodulin
V-A: Veno-arterial
VEGF: Vascular endothelial growth factor
V-V: Veno-venous
